# Should the next South African census be register-based?

**DOI:** 10.2471/BLT.14.148502

**Published:** 2015-03-01

**Authors:** Sulaiman Bah

**Affiliations:** a Health Information Management and Technology, University of Dammam, PO Box 2435, Dammam, 31441, Saudi Arabia.

All governments need detailed information on their populations to design and run their health systems effectively. Most countries collect a large part of these data through a census; an exercise often held once every decade that aims to count every inhabitant of every household. With growing populations, the census becomes progressively more difficult and costly. Some countries are moving towards an alternative; combining data from existing population-based registers – such as those that collect births, deaths and addresses – with data from other registers, such as employment and education.

A census based on an administrative data register offers several advantages. Once established, a register-based census can be relatively inexpensive and rapid, and reduce the need for respondents to report the same information to different government departments. A handful of high-income countries – Bahrain, Denmark, Finland, Norway and Singapore – have conducted register-based censuses and other governments are currently debating the feasibility of adapting their administrative data registers for this purpose.^1^

As an upper-middle income country of 53 million people, South Africa has a well-established door-to-door method of enumeration. The 1996 and 2001 national censuses in South Africa – which were both based on door-to-door household visits – played an important role in the development of the country’s post-apartheid identity. Each of these censuses led to the employment – albeit often short-term – of approximately 100 000 individuals. In addition, door-to-door visits represented the only feasible method of enumerating most rural South Africans in the 1990s.

There are at least five reasons why register-based censuses should now look attractive to the South African government. First, they should markedly reduce costs. The personnel and professional services’ costs for each South African census rose from 437 million rand (37.7 million United States dollars) in 1996 to 625 million in 2001^2^ and over 1 billion in 2011.^3^ Second, almost all South Africans are now recorded in the national population register, which was set up more than 60 years ago. In 2012 and 2014, respectively, the national statistical authority – Statistics South Africa – estimated that this register covered about 90% of births^4^ and 94% of deaths among South Africans aged at least 15 years.^5^ Since 2009, there has been an on-going campaign – partly driven and facilitated by improved information technology – to clean and improve the population register. Third, South Africa also has a comprehensive business register that is functional, effective and continually improving. Fourth, geographical information systems – which can play an important role in the development of land and property registers – have already been used in three censuses in South Africa and have become increasingly useful. Fifth, Statistics South Africa has already set up a national statistics system that coordinates the production of official statistics from many different registers and national sources. This system also includes an operational framework for the assessment of statistical quality.

It is clear, from the experiences of those countries that have already implemented a register-based census, that the transition from a traditional census – with door-to-door visits –is seldom smooth. Quality assessment, compatibility and interoperability appear to be particular challenges. A country may have several relevant registers that overlap in the information they hold. Data quality and storage formats may differ. It may be difficult and costly to transfer data to a census database and to a final data pool, especially while maintaining data privacy. Logical rules need to be put in place to address any conflicts between the attributes in different registers. A legislative framework and public support for the use of these data are needed.

The journey towards a first register-based census takes a long time. South Africa – with its fairly comprehensive population register, its relatively advanced systems for electronic governance and its national statistics system – is, potentially, already close to the end of that journey. The South African government is now in a position to consider future use of a register-based census.
